# Electronic, Optical, and Vibrational Properties of an AgAlS_2_ Crystal in a High-Pressure Phase

**DOI:** 10.3390/ma16217017

**Published:** 2023-11-02

**Authors:** Myron Ya. Rudysh, Anatolii O. Fedorchuk, Mikhail G. Brik, Jurij Grechenkov, Dmitry Bocharov, Sergei Piskunov, Anatoli I. Popov, Michal Piasecki

**Affiliations:** 1 Department of Theoretical Physics, Jan Dlugosz University in Częstochowa, 13/15, Armii Krajowej Al., 42-200 Częstochowa, Poland; m.piasecki@ujd.edu.pl; 2 Department of Inorganic and Organic Chemistry, Lviv National University of Veterinary Medicine and Biotechnologies, Pekarska Str. 50, 79010 Lviv, Ukraine; ft@ua.fm; 3 School of Optoelectronic Engineering & CQUPT-BUL Innovation Institute, Chongqing University of Posts and Telecommunications, Chongqing 400065, China; 4 Centre of Excellence for Photoconversion, Vinča Institute of Nuclear Sciences—National Institute of the Republic of Serbia, University of Belgrade, 11001 Belgrade, Serbia; 5 Institute of Physics, University of Tartu, 50411 Tartu, Estonia; grecenkovs@cfi.lu.lv; 6 Institute of Solid State Physics, University of Latvia, Kengaraga 8, LV-1063 Riga, Latvia; bocharov@cfi.lu.lv (D.B.); piskunov@cfi.lu.lv (S.P.); popov@latnet.lv (A.I.P.); 7 Academy of Romanian Scientists, Ilfov Str. No. 3, 030167 Bucharest, Romania; 8 Transport and Telecommunication Institute, LV-1019 Riga, Latvia

**Keywords:** crystal, phase transition, CASTEP, density functional theory, phonons

## Abstract

The aim of this study is to comprehensively examine the structural composition and properties of the AgAlS_2_ crystal during its high-pressure phase. This analysis delves into the second coordination environment of the crystal structure and elucidates the distinct transformations it undergoes during the phase transition. The band energy structure was calculated, and the origin of electronic levels was clarified. It is shown that the crystal becomes non-stratified during the phase transition. This study also determined the values of the crystal’s carrier effective masses, underscoring its spatial anisotropy. It was found that the calculated optical functions are similar to the crystal in the chalcopyrite structure, and their differences are shown. Further, this study involved the calculation of the crystal’s phonon spectrum, revealing the spectrum’s transformation during the phase transition. The vibrational frequencies were also obtained, with a symmetrical classification of vibrational modes. Finally, this study derived the infrared and Raman spectra of the AgAlS_2_ crystal, thereby providing a comprehensive picture of the crystal during its high-pressure phase.

## 1. Introduction

Ternary semiconductor materials, known for their diverse structures and physical properties, are increasingly gaining traction in the scientific community due to their optimally ranged band gap, making them suitable for various electronic applications [1,2]. These materials include crystals from the group I-III-VI_2_ (I = Cu, Ag, III = Al, Ga, In, VI = S, Se, and Te), which typically possess a chalcopyrite structure under normal conditions. [3]. This structure, lacking a center of symmetry, opens the possibility for non-linear optical and piezoelectric effects. Ternary chalcopyrite semiconducting compounds, which can be represented by the general formula I-III-VI_2_, have gained significant attention recently due to their various applications, such as photocathodes for water splitting and non-linear optical (NLO) devices [4,5]. Certain I-III-VI_2_ family compounds, such as AgAlS_2_, AgAlSe_2_, and AgAlTe_2_, have displayed remarkable NLO properties and are considered promising photocathodes for water splitting and photovoltaic detectors. In addition, these materials are attractive from the point of view of their use as an absorbing layer in photovoltaic cells. A high absorption coefficient and a band gap close to the optimum allow the use of these materials for thin-film photovoltaic cells with an efficiency of more than 24%. In a wider scope, silver-containing materials were studied for their antibacterial properties [6] and electrochemical applications [7].

Our earlier studies have explored several ternary chalcogenide compounds, including AgGaTe_2_, AgGaS_2_, AgAlS_2_, and Ag_2_SiS_3_ [8,9,10,11,12]. In the conducted comprehensive theoretical first-principle investigations, we focused on understanding their various properties, such as electronic structure, optical behavior, and elasticity. Additionally, we explored the impact of isomorphic substitution within the CuGa(S*_x_*Se_1−*x*_)_2_ system on the optical, electronic, and elastic characteristics, and found a possibility for adjusting material parameters accordingly, as reported in Ref. [13].

Other first-principle studies also focused on similar compounds, with a focus mainly on the tetragonal phase of the respective crystal [14]. In [15], the vibrational properties of various silver-containing chalcopyrites under pressure were studied, and in [16], the authors study the effect of axial stress on AgGaS_2_.

Understanding the structural and property changes during phase transitions is crucial for elucidating the material’s application scopes. Our current study examines the transformation of the electronic spectrum of the AgAlS_2_ crystal during a high-pressure phase transition. The AgAlS_2_ crystal is a typical representative of the I-III-VI_2_ group, which crystallizes with a lattice of the chalcopyrite type. Despite limited studies on the structure and properties of this material, it is known to have a direct band gap of 3.13 eV [17] and to undergo phase transition at a hydrostatic pressure of 25 kbar [18]. Under normal conditions, the crystal belongs to the tetragonal structure of the chalcopyrite type with lattice parameters *a* = 5.6950 Å and *c* = 10.2600 Å and cell volume *V* = 332.80 Å^3^ [17]. The crystal has two types of deformation of the crystal lattice. The first corresponds to the displacement of the anion in the *xy* plane relative to its ideal position and is *u* = 0.298. The second deformation is tetrahedral and is η = 0.901. At high pressures, the crystal transitions to trigonal symmetry *P*3*m*1 (space group no. 156) with lattice parameters *a* = 3.5 Å, *b* = 3.5 Å, and *c* = 6.84 Å and cell volume *V* = 72.564 Å^3^ [18]. Phase transitions have also been observed in other crystals of this group. At 3–4 Gpa, AgInTe_2_ transforms into a cation-disordered orthorhombic structure with space group *Cmcm* [19], as well as 12.7 GPa for CuGaTe_2_ and at 3.6 GPa for the CuInTe_2_ crystal and others [20,21,22,23,24].

The present research aims to theoretically explore the electronic, optical, and vibrational characteristics of the high-pressure phase of the AgAlS_2_ crystal. Earlier in the paper [11], an AgAlS_2_ crystal with a chalcopyrite structure was studied under the influence of hydrostatic pressures. This work aims to investigate the transformation of the crystal’s properties during the phase transition to a phase with trigonal symmetry.

The structure of this manuscript unfolds as follows. Section 2 provides a detailed description of the computational methodologies employed in this research. Subsequently, Section 3 presents an in-depth analysis and discussion of the calculated structural properties of the AgAlS_2_ compound, the band structure and the chemical, optical, and vibrational properties of the AgAlS_2_ in its high-pressure phase. Section 4 overviews the key findings, and, finally, Section 5 describes conclusions derived from this study.

## 2. Materials and Methods

In the work, the density functional theory (DFT) method was used to study the properties of the AgAlS_2_ crystal from the first principles. Calculations of electronic structure, optical properties, phonon band structure, and other vibrational properties have been calculated using the CASTEP (CAmbridge Serial Total Energy Package) code (DFT) [25]. The method is based on self-consistent solving of the Kohn–Sham equations. Convergence of the self-alignment cycle was achieved in 42 iterations, which resulted in a total crystal energy of −3950.72 eV. The effects of exchange correlation were considered using the Generalized Gradient Approximation (GGA) with the Perdue–Burke–Ernzerhoff (PBE) parameterization [26]. This method is optimal in terms of computational cost and the quality of the obtained results. In particular, it has demonstrated good performance in describing vibrational modes [6,7], elastic properties [11], and optical spectra [7] of similar-structured and -composed ternary chalcogenides. Therefore, the utilization of this function for the AgAlS_2_ crystal in the high-pressure phase is justified. The electron–ion interaction was described using the norm-conserving pseudopotential NCPP [27]. Pseudoatomic calculations were performed for Ag 4*s*^2^ 4*p*^6^ 4*d*^10^ 5*s*^1^ and Al 3*s*^2^ 3*p*^1^; S 3*s*^2^ 3*p*^4^. The plane wave basis set cut-off was taken to be equal to *E*_cut_ = 850 eV. The integration over the first Brillouin zone was carried out on a *k*-mesh, which was selected as 5 × 5 × 2 using the Monkhorst–Pack method [28]. Initial data for the calculations were derived from a previous study of the crystal’s structure [18]. Prior to the calculations, the crystal’s unit cell underwent optimization, which entailed the adjustment of the crystal lattice parameters and the relative coordinates of the atoms. A Broyden–Flatcher–Goldfarb–Shanno (BFGS) algorithm was applied for geometric optimization within fixed symmetry constraints [29]. For crystal structure optimization, we used the following convergence criteria: maximal force −1 × 10^−2^ GPa, maximal displacement −5 × 10^−5^ Å, and energy convergence 5 × 10^−5^ eV/atom.

## 3. Results

### 3.1. Structure of AgAlS_2_ Crystal

**Figure 1:**
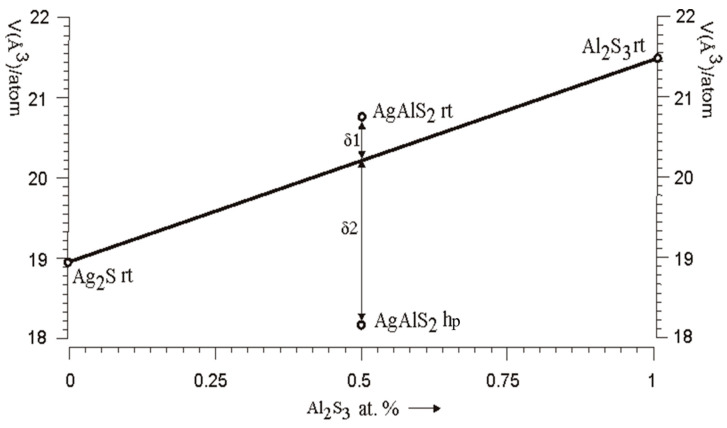
Average experimental atomic volume per compound atom vs. Al_2_S_3_ content for AgAlS_2_ mixed compounds in the system Ag_2_S (left side)-Al_2_S_3_ (right side).

**Figure 2:**
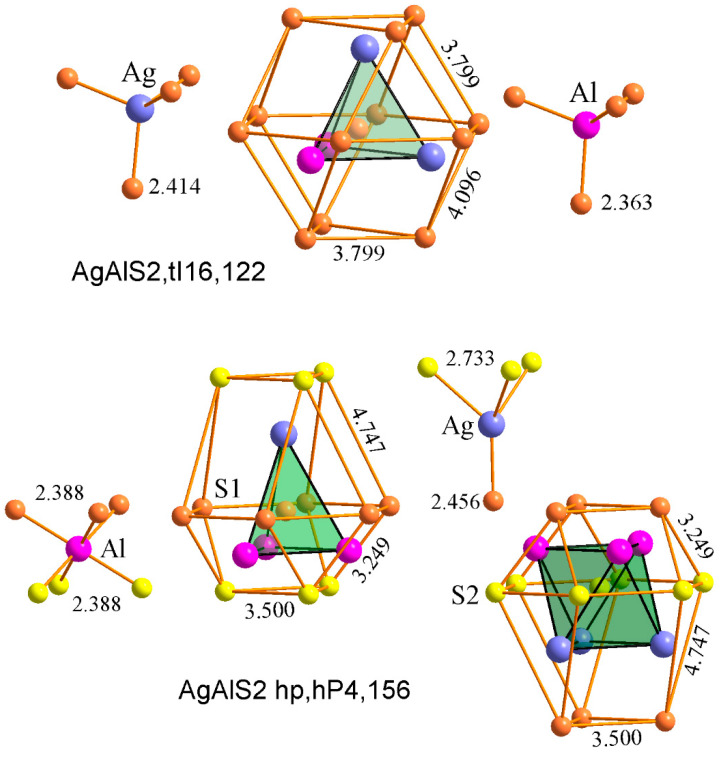
The closest and second coordination environment of sulfur atoms (S1—orange; S2—yellow) and interatomic distances to Ag (blue) and Al (magenta) atoms in the structure of compounds AgAlS_2_ rt, tI16,122 and AgAlS_2_ hp, hP4,156.

**Figure 3:**
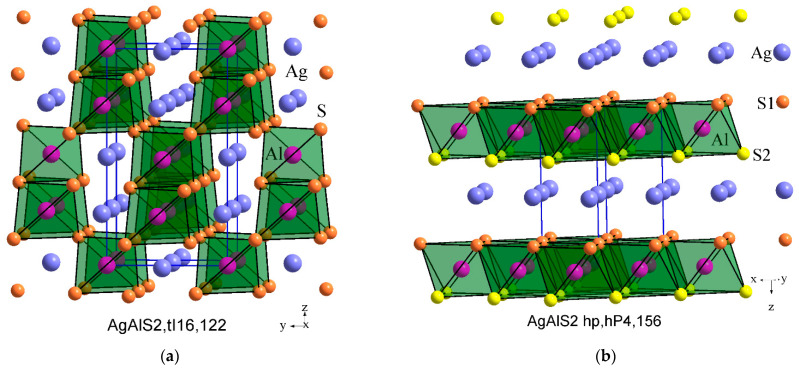
Stacking of polyhedra from sulfur (S1—orange, S2—yellow) atoms around aluminum atoms (magenta) in the structure of compounds AgAlS_2_,tI16,122 (**a**) and AgAlS_2_ hp,hP4,156 (**b**). Ag atoms are shown with blue color.

The AgAlS_2_ compound is known to exist in two structures. The first structure of AgAlS_2_ at room temperature (AgAlS_2_,tI16,122) is observed under normal conditions and possesses a tetragonal symmetry with a chalcopyrite-type cell. The second phase (II), denoted as AgAlS_2_ ph (AgAlS_2_ hp,hP4,156), exists at high pressures. The compounds AgAlS_2_ rt [18] and AgAlS_2_ hp [18] can be considered as the result of the interaction of binary sulfides Ag_2_S rt (Ag_2_S mP12,14) [30] and Al_2_S_3_ rt (Al_2_S_3_ hP30,169) [31]. The unit cell volume, when measured experimentally and adjusted to the number of atoms in the unit cell, can indirectly reflect the interaction of atoms in these compounds. As illustrated in Figure 1, average atomic volume deviates from Vegard’s rule in different ways for compounds AgAlS_2_ rt and AgAlS_2_ hp, which may indicate some difference in the nature of the interaction of components in these compounds. Specifically, for the compound AgAlS_2_ rt, there is a slight increase (δ1), and for the compound AgAlS_2_ hp, there is a significant (δ2) decrease in the average value of the atomic volume relative to the Vegard line. As can be seen in Figure 2, the nearest coordination environment (SCE) of sulfur atoms is in the form of a cubooctahedron for AgAlS_2_ rt, tI16,122 and is in the form of a hexagonal analog of a cubooctahedron for AgAlS_2_ hp, hP4,156. This may indicate a predominantly ionic type of chemical bond in these ternary compounds. However, under the influence of static pressure, the type of ionic sublattice changes from the sphalerite type [32] for AgAlS_2_ rt to the wurtzite type in the case of AgAlS_2_ hp. At the same time, aluminum atoms shift from tetrahedral cavities to octahedral ones. The compound AgAlS_2_ rt (Figure 3) can be represented as a framework of tetrahedra connected by vertices formed by sulfur atoms around aluminum atoms. Silver atoms occupy the channels in the frame while having a symmetric (located in the centers of tetrahedra) tetrahedral environment (Figure 2). In contrast, the AgAlS_2_ hp compound can be represented as interconnected blocks of octahedra, formed by sulfur atoms around aluminum atoms and connected by edges. As illustrated in Figure 3, the distance between the blocks for the AgAlS_2_ hp compound is relaxed, and the atoms in the tetrahedral environment are displaced from the centers. This structure may be sensitive to temperature changes, which will be manifested in significant thermal fluctuations of heavy silver atoms within the light anionic sublattice, which is of interest to thermoelectrics. The compound AgAlS_2_ hp can be considered layered, and, therefore, it has possible properties that are characteristic of layered structures. Such a drastic change in the crystal structure during the phase transition can indicate a sharp variation in the physical properties within the phase transition region of materials based on these alloys, potentially making them suitable as sensors for temperature/pressure changes within the transition region.

In this paper, before calculating the physical properties of the AgAlS_2_ crystal in the phase under hydrostatic pressure, geometric optimization of its crystal structure was carried out by the BFGS method. The optimized parameters of the lattice are given in Table 1, and the fractional coordinates of the atoms are given in Table 2. As can be seen in Table 1, the lattice parameters optimized using the GGA-PBE function are slightly larger than the experimental ones. The overestimation of lattice parameters is 0.032Å, for a and b parameters, and 0.006 Å, which is 0.91% and 0.08%, respectively. This excellent alignment between the theoretical and experimental lattice parameters suggests that the chosen methodology effectively captures the characteristics of the studied compound. The deviation of the volume of the unit cell of the crystal is δ*V* = 1.386 Å^3^, which is 1.91%. The small deviations of the lattice constant values are well inside the acceptable value range, and the overestimation can be attributed to various factors, e.g., the self-interaction error that leads to the widening of the wave function and is not explicitly accounted for in our methodology.

### 3.2. Formatting of Mathematical Components

Additionally, we calculated the relative change in the average unit cell deviation using the following formula:

(1) $$d_{r}=\frac{V_{c}^{\left(\mathrm{opt}\right)}{}^{1/3}-V_{c}^{(\exp)1/3}}{V_{c}^{(\exp)1/3}}.$$

Here, $V_{c}^{\left(\exp\right)}$ represents the experimentally obtained unit cell volume, while $V_{c}^{\left(\mathrm{opt}\right)}$ denotes the calculated (optimized) unit cell volumes. This value can serve as an indicator of the deviation between the optimized structure and the experimental one. The obtained unit cell deviation parameter is equal to 0.0063, which is a very small value, confirming a consistency of the experimental and calculated structures (deviation ~0.6%). Table 2 (last column) collects the distances between the calculated (optimized) and experimental fractional atomic coordinates *z/c*.

### 3.3. Band Structure and Chemical Bond Analysis

**Figure 4:**
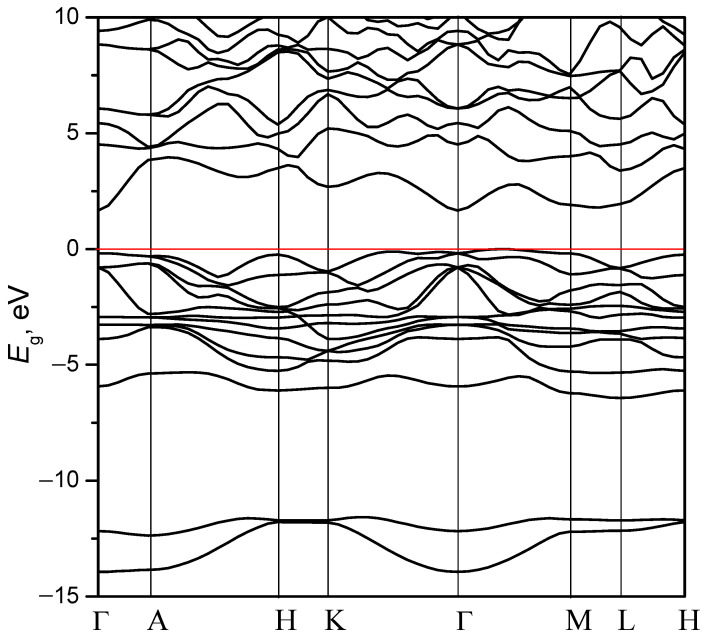
Calculated band structure of the AgAlS_2_ crystal in the high-pressure phase. The coordinates of the high symmetry points are the following: Г (0, 0, 0); A (0, 0, 0.5); H (−0.333, 0.667, 0.5); K (−0.333, 0.667, 0); M (0, 0.5, 0); L (0, 0.5, 0). The coordinates of the high symmetry points were automatically generated by the CASTEP code. Red line marks the Fermi level.

For the studied crystal, we constructed the band energy structure for the crystal at high symmetry points in the Brillouin zone and along the lines connecting them. The following sequence of points was used to construct the zone diagram: Г → A → H → K → Г → M → L → H. The coordinates of the corresponding points in the Brillouin zone automatically generated by the CASTEP code are given in the caption of Figure 4. Figure 4 illustrates the band energy structure calculated for the AgAlS_2_ crystal in the high-pressure phase. For the crystal with trigonal symmetry, the top of the valence band is located on the segment Г–M near the point Г. The bottom of the conduction zone is located in the center of the Brillouin zone (point Г), which indicates an indirect band gap type. It is also notable that the local minimum of the bottom of the conduction band is located on the segment M–L, which is 0.13 eV higher than the position of the level at point Г. The calculated smallest band gap is *E_g_* = 1.67 eV (indirect), corresponding to the M → Г transition. The direct band gap in the Brillouin zone center corresponds to the higher value of energy (*E_g_* = 1.84 eV). If we consider the AgAlS_2_ crystal in a chalcopyrite structure, the band gap is direct [33,34], and the band gap value is equal to 3.13 eV. The calculated band gap value using the GGA-PBE function is equal to 1.99 eV [11] and is larger than a crystal in trigonal symmetry. This reveals that the transition to trigonal symmetry leads to direct-to-indirect band gap transformation and a slight decrease of *E_g_*. We also expected that the experimental value of the band gap for the crystal in the high-pressure phase is approximately 1–1.2 eV higher than the calculated value. Thus, for absorption in the crystal with energy corresponding to transitions between the top of the valence band and the bottom of the conduction band, phonon participation is a necessity.

As can be seen in the figure, the band structure comprises two subbands that form the valence band of the crystal. The conduction band, on the other hand, is formed by a broad band that encompasses a large number of electronic levels. Both the conduction and valence bands demonstrate substantial dispersion, although the highest level of the valence band shows relatively less dispersion. In the interval −6–−12 eV, an energy band gap is observed.

The band structure provides insight into the properties of charge carriers within the material. In particular, the curvature of electronic levels near the extremum is related to the effective mass of charge carriers. The relationship between the effective mass and the band structure is represented by the following equation:

(2) $${\left(\frac{1}{m*}\right)}_{ij}=\frac{1}{\hbar^{2}}\frac{d^{2}E_{n}(k)}{dk_{i}k_{j}},$$

where m* is the effective mass, ℏ the reduced Planck constant, *I* and *j* represent the *x*, *y*, and *z* directions in the reciprocal space, and *E_n_*(*k*) is the dispersion relation for the *n*^th^ electronic band. The effective mass is calculated from the approximation of the electronic level near the extremum by a parabolic equation. Thus, the effective mass of the hole m_h_* is obtained for the level of the valence band, and the effective mass of the electron m_e_* is obtained for the lowest level of the conduction band.

**Figure 5:**
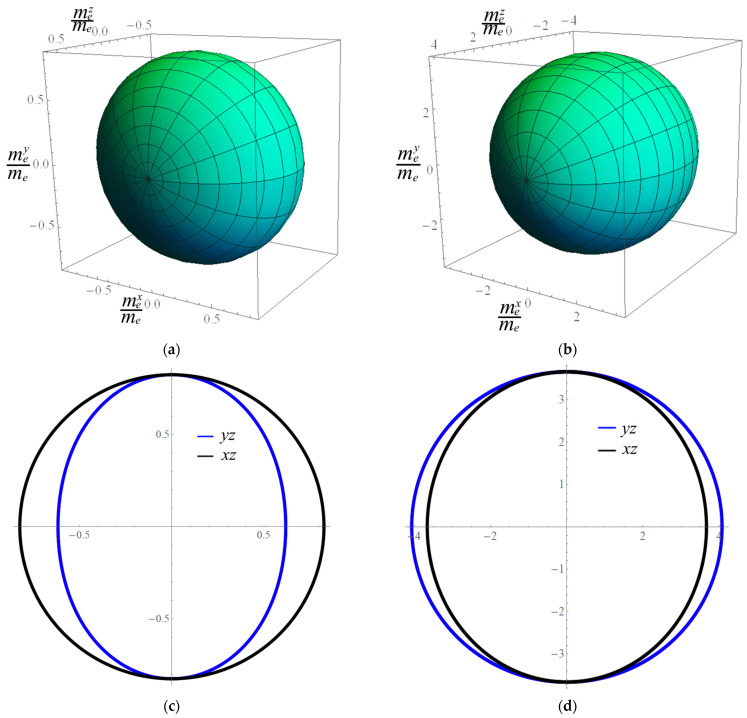
Three-dimensional contour plots of the holes (**a**), electron (**b**) effective masses, and corresponding projections on *xz* and *yz* planes for the hole (**c**) and electron (**d**), respectively.

Generally, the effective mass is anisotropic, which is characterized by different curvature of the energy levels of the electron in different directions of the k-space and is expressed in units of the mass of a free electron $m_{x}^{*}=\frac{m_{x}}{m_{e}}$, $m_{y}^{*}=\frac{m_{y}}{m_{e}}$, $m_{z}^{*}=\frac{m_{z}}{m_{e}}$. These masses correspond to the directions [100], [010], and [001]. The AgAlS_2_ crystal in phase II has two independent directions and, accordingly, the components of the elective mass of charge carriers are as follows: $m_{x}^{*}=m_{y}^{*}\neq m_{z}^{*}$. The calculated values of effective masses of holes and electrons are as follows. For the *x* direction, m_e_* = 0.824 and m_h_* = 3.661, and for the *z* direction, m_e_* = 0.617 and m_h_* = 4.069 (all values are given in *m_e_* units). It is clear that the two components vary due to the material’s anisotropy, yielding ratios of m_x_*/m_z_* = 1.33 for electrons and 0.899 for holes. To visualize this anisotropy, we depicted the spatial distribution of the effective mass of charge carriers (Figure 5). As can be seen in the figure, the spatial distribution of the effective mass of electrons in the AgAlS_2_ crystal has greater anisotropy than the effective mass of the holes. Thus, the electron’s effective mass exhibits significant compression in the *z*-direction, while the spatial distribution of the hole’s effective mass reveals an elongation along the same direction. The obtained results are consistent with the mass ratio in the corresponding directions.

**Figure 6:**
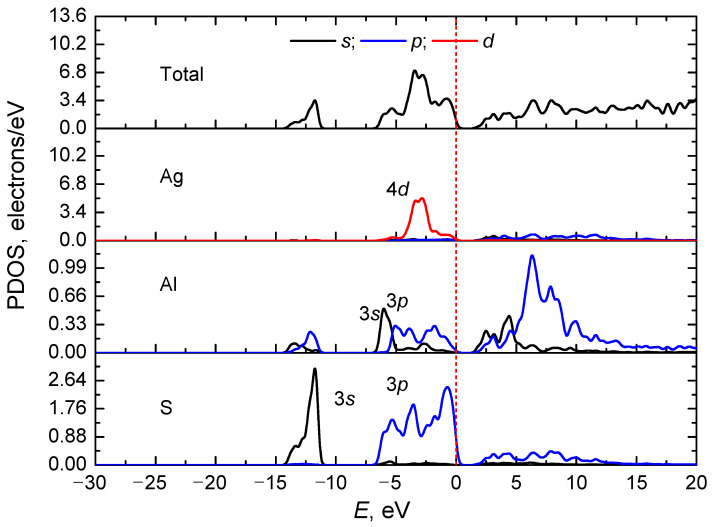
Total and partial (for Ag, Al, and S atoms) density of states *N(E)* for the AgAlS_2_ crystal in the high-pressure phase calculated using the GGA-PBE function. The red dotted line marks the Fermi level.

A more detailed analysis of the electronic spectrum can be carried out by calculating the total and partial densities of the electronic states of the AgAlS_2_. Figure 6 shows the full and partial density of electronic states of the AgAlS_2_ crystal within the high-pressure trigonal phase (phase II). In the figure, it is discernible that four subbands are formed by groups of electronic levels within the energy range −30–0 eV. The first subgroup forms the top of the valence band of the crystal and lies in the energy range of −6.5–0 eV. It is mainly formed by 3*p*-states of sulfur intermixed with impurities of 3*p*-states of aluminum. Ag 4*d*-electron states are situated in the middle of this range, while the lowest levels of this subband are formed by Al 3*s*-states. A sublevel positioned slightly lower −13 eV is formed by the 3*s*-states of sulfur together with a mixture of aluminum 3*s*- and 3*p*-states.

The conduction band is formed by a collection of electronic states of different types corresponding to different atoms. The bottom of the conduction band is formed by the *s*-states of Al atoms, while higher energies correspond to the combination of the *p*-states of S atoms with the *p*-states of Al atoms.

Broadly speaking, for the AgAlS_2_ crystal, the total and partial density of electronic states do not differ significantly from the crystal in the chalcopyrite structure. The most significant effect of the transformation of the crystal lattice during the phase transition is observed for 4*d*-states of Ag atoms, which is characterized by a decrease in the intensity of the band at energies of approximately −2.5 eV [11]. Effective charges of all atoms are given in Table 3.

To additionally analyze the type of bonding, we calculated the ionicity of bonds using the following equation:

(3) $$f_{h}=1-e^{-\frac{\left|P_{c}-P\right|}{P}},$$

In this equation, *P* is the overlap population and *P_c_* is the overlap population of purely covalent crystals and is equal to 1. The equality of the *f_h_* parameter to 1 indicates an ionic bond type, while a zero value indicates a purely covalent bond type. The calculated bond ionicity values are given in Table 4. As can be seen in the table, the crystal has a covalent type of chemical bonding with a large contribution of the ionic component. For the AgAlS_2_ crystal in the high-pressure phase, Al–S bonds have a significant share of covalency. At the same time, Ag–S bonds are characterized by a considerable share of the ionic component of the bond. Compared to chalcopyrite, the studied crystal is less covalent (for chalcopyrite structure for Al–S *f_h_* = 0.487 and Ag–S *f_h_* = 0.958).

Compared with the crystal in the structure of chalcopyrite, it can be noted that in phase II, the bond lengths of both Al–S and Ag–S are slightly longer, which is related to the layering of the crystal structure. The calculated value of the ionicity of the chemical bond indicates that the Al–S bonds have a strong covalent bond. However, for Ag–S atoms, the interaction has an ionic character with an insignificant covalency contribution, which is consistent with the crystal–chemical analysis of the structure and the second coordination environment.

### 3.4. Optical Properties

The optical properties of crystals, along with other characteristics, determine their potential for practical applications as optical materials and various control and detection devices. In particular, the amount of absorption, the location and width of the transparency window, and the value and dispersion of the refractive indices, as well as their anisotropy, are crucial for evaluating the perspective of material usage.

Optical spectra of materials can be obtained using the results of first-principle calculations. In particular, the dielectric function can provide important information about the optical characteristics of materials and related optical spectra such as reflection spectra *R*(ω), refractive index *n*(ω), absorption coefficient α(ω), etc. The dielectric function ε(ω) is a complex function and can be written as ε(ω) = ε_1_(ω) + *i*ε_2_(ω), where ε_1_(ω) and ε_2_(ω) are its real and imaginary parts, respectively. The imaginary part of the dielectric function ε_2_(ω) = Im(ε) is directly related to light absorption processes. It can be estimated by the integration of elements of the dipole matrix operator between the filled states of the valence band and empty levels of the conduction band [35]:

(4) $$\varepsilon_{2}(\hbar \omega )=\frac{2\pi e^{2}}{{\Omega \varepsilon }_{0}}{\sum_{k,v,c}\left|\langle \psi_{k}^{c}\left|ur\right|\psi_{k}^{v}\rangle \right|}^{2}\delta \left(E_{k}^{c}-E_{k}^{v}-E\right)$$

where *e* is the electron charge, Ω is the unit cell volume, $\psi_{k}^{c}$ and $\psi_{k}^{v}$ are the wave functions of the conduction band and valence band in *k*-space, respectively, ε_0_ is the dielectric permittivity of vacuum, *u* is the incident photon polarization vector, **r** is the operator of electron position, and *E* is the photon energy. The excited states are represented as unoccupied Kohn–Sham states. The matrix elements of position and momentum operators are related [36]:

(5) $$\langle \psi_{k}^{c}|r|\psi_{k}^{v}\rangle =\frac{1}{i\omega m}\langle \psi_{k}^{c}|p|\psi_{k}^{v}\rangle +\frac{1}{\hbar \omega }\langle \psi_{k}^{c}(|V_{nl}|,r)\psi_{k}^{v}\rangle ,$$

where *p* represents the momentum operator, *m* is the mass of the electron, ℏω is the energy difference between occupied and unoccupied energy levels, and *V_nl_* is the non-local pseudopotential (angular momentum-dependent potentials). 

The real part of the dielectric function ε_1_(ℏω) = Re(ε) is related to its imaginary part ε_2_(ℏω) and can be acquired using the Kramers–Kronig transformation:

(6) $$\varepsilon_{1}(\hbar \omega )=1+\frac{2}{\pi }\int_{0}^{\infty }\frac{t\varepsilon_{2}(t)dt}{t^{2}-{(\hbar \omega )}^{2}}.$$

**Figure 7:**
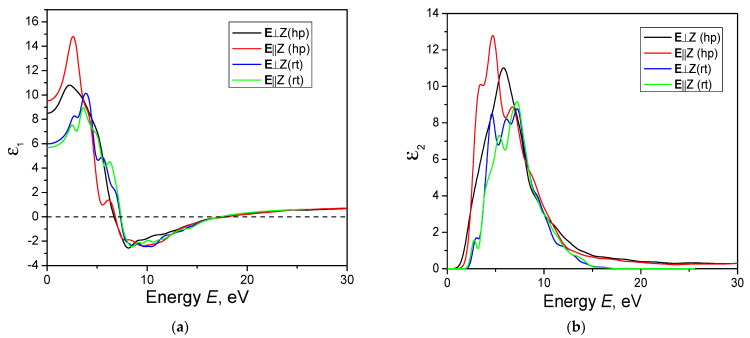
Real (**a**) and imaginary (**b**) parts of the dielectric function calculated for the AgAlS_2_ crystal at the high-pressure phase (ph) and room temperature phase (rt) using the GGA-PBE function.

For the optical spectra, instrumental smearing 0.2 eV (instead of 0.5 eV) was used to model broadening effects. This value was used for reducing peak broadening. Figure 7 shows the spectra of the real and imaginary dielectric function of the AgAlS_2_ crystal in the hp phase calculated using the GGA-PBE function. The symmetry of the crystal lattice determines that there are two independent directions for the AgAlS_2_ crystal, and the dielectric function is determined by the components ε*_x_* = ε*_y_* and ε*_z_*., We calculated the dielectric function for ε_x_ (**E**⊥*Z*) and ε*_z_* (**E**||*Z*) for this paper. As can be seen in Figure 7a, the dielectric function increases smoothly, starting from the energy value of 0 to 2.6 eV, followed by a swift decrease. At an energy of 6.7 eV, the real part of the dielectric function turns zero and becomes negative with a further increase in energy.

The static dielectric function Is represented by a set of closely spaced overlapping bands. The imaginary part of the dielectric function is related to the photon absorption process. The first peak at the lowest energy corresponds to optical absorption, which is related to the fundamental absorption in the crystal. The following bands characterize band–band transitions in a crystal at a level with higher energy than the band gap value.

At low energies, the dielectric function exhibits substantial anisotropy, which is accompanied by a significant difference in values in different directions. This trend is observed for both real and imaginary parts of the dielectric function. The greatest anisotropy of the static dielectric function is observed at its maximum value. At energies larger than about 8 eV, the anisotropy decreases sharply and approaches zero. The values of the static dielectric function are 8.5 and 9.5 for the **E**⊥*Z* and **E**||*Z* directions, respectively, while for a crystal with a chalcopyrite structure, they are 5.98 and 5.68 for **E**⊥*Z* and **E**||*Z*, respectively [11]. To numerically estimate the anisotropy of the dielectric function, we used the formula for uniaxial anisotropy:

(7) $$\delta \varepsilon =\frac{\varepsilon_{0}^{z}-\varepsilon_{0}^{x}}{\varepsilon_{0}^{tot}},$$

where $\varepsilon_{0}^{z}$ and $\varepsilon_{0}^{x}$ are the static dielectric constants and $\varepsilon_{0}^{tot}$ is the total dielectric constant. The calculated value of uniaxial anisotropy of the dielectric function for AgAlS_2_ crystals at high pressures is equal to δε = 0.055. 

Taking into account the results of the calculation of the band energy structure and densities of electronic states, we infer that the fundamental absorption edge of the studied crystal is formed by optical transitions from p-states of S atoms at the top of the valence band to s-states of Al atoms at the bottom of the conducting band.

In Figure 7, the spectrum of the dielectric function shown has a similar structure to the AgAlS_2_ crystal in chalcopyrite structure. A slight increase in the dielectric function in the high-pressure phase is observed. The absence of a well-defined peak at energies slightly higher than E_g_ obtained for the chalcopyrite crystal structure [11] can also be attributed to impurities.

Using the following expressions, the refractive index n and extinction coefficient k spectra can be obtained:

(8) nℏω=εℏω12+εℏω2212+εℏω12,kℏω=εℏω12+εℏω2212−εℏω12.

**Figure 8:**
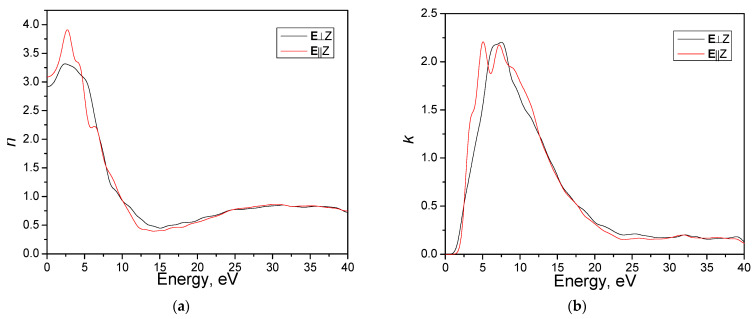
Calculated refractive index *n* (**a**) and extinction coefficient *k* (**b**) for the AgAlS_2_ crystal at the hp phase obtained using the GGA-PBE function.

Figure 8 shows the calculated spectra n(ℏω) and k(ℏω). The refractive index spectrum and the extinction coefficient, as depicted in Figure 8, have similar characteristics as the dielectric function spectrum. The static values of the refractive index are 2.92 and 3.08 for the **E**⊥Z and **E**||Z directions, respectively.

**Figure 9:**
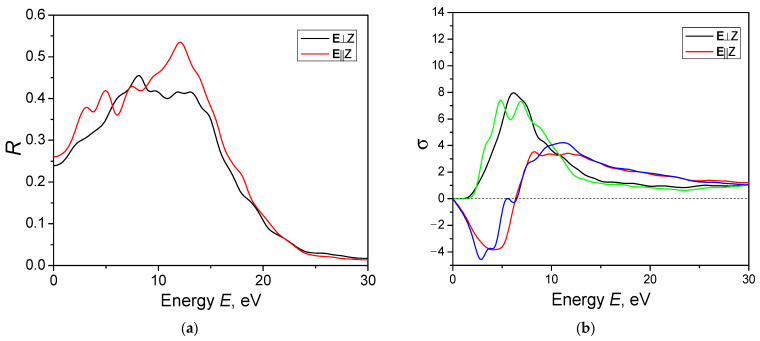

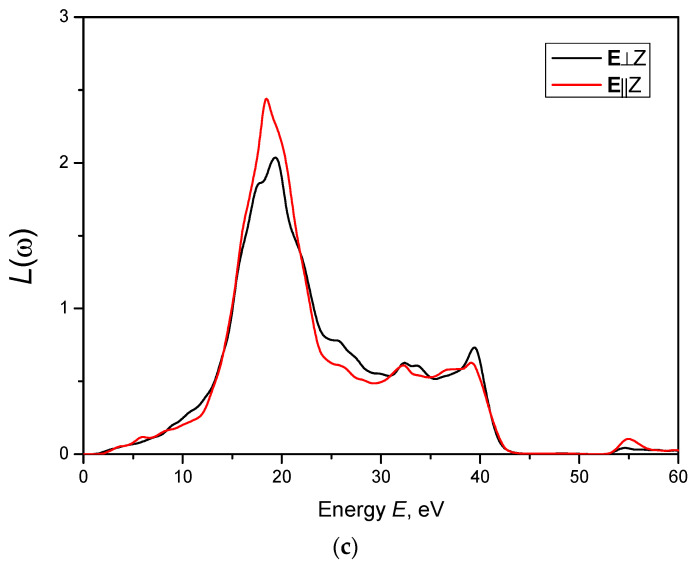
Calculated optical function: (**a**) reflectivity; (**b**) optical conductivity; (**c**) loss function.

Figure 9 shows the reflection spectrum of the AgAlS_2_ crystal in phase II. As the figure illustrates, the average value of the reflection coefficient is approximately 0.4. As the energy increases from 0 to 12 eV, the reflection coefficient increases, peaking at R = 0.53. As energies became larger than 12 eV, the reflection coefficient decreased monotonically. At low energy values, there is significant anisotropy in the reflection spectrum. Upon reaching the maximum value, total anisotropy reduces. Figure 9b,c also show the optical conductivity spectrum and loss function.

It is important to consider that the calculation of optical spectra carried out in the work does not take into account a number of factors, such as the contribution of phonons to optical spectra, which is important for indirect band crystals. Unfortunately, no literature data exists regarding experimental studies of optical spectra, so the results obtained in this work remain purely theoretical predictions.

### 3.5. Vibrational Properties of AgAlS_2_ in II the High-Pressure Phase

The study of the vibrational spectrum of the AgAlS_2_ crystal in its chalcopyrite phase was carried out in [12]. It is known that during phase transitions, both the crystal structure and various physical properties change. In particular, when the structure of the crystal changes during the phase transition, the symmetry of the crystal lattice changes, and, as a result, the structure of the vibrational spectrum also undergoes changes. Consequently, we calculated the vibrational spectra of the AgAlS_2_ crystal in its high-pressure phase, also known as phase II, using density functional perturbation theory (DFPT). The AgAlS_2_ crystal belongs to the *P*3*m*1 space group, which is homomorphic to the point symmetry group 3*m*1 (*C*_3*v*_). This symmetry group contains six symmetry elements. The table of characters of this point group is shown in Table 5.

**Figure 10:**
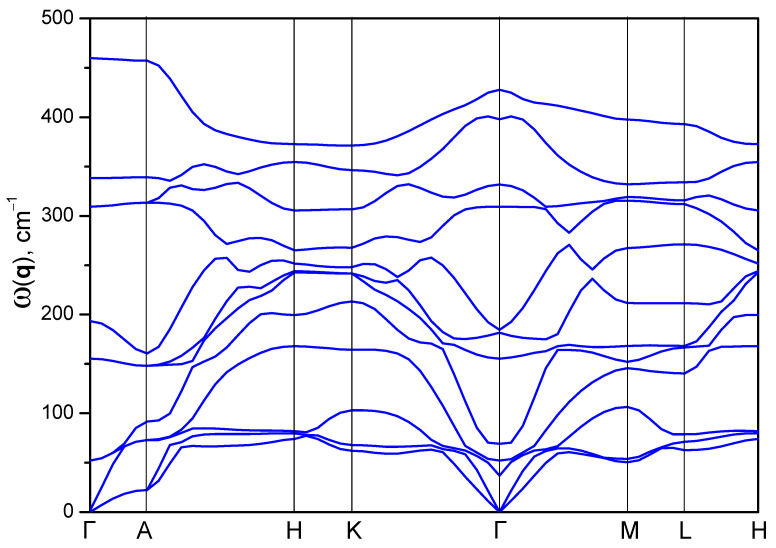
Calculated phonon dispersion of the AgAlS_2_ crystal in the high-pressure phase.

As a result of the group-theoretic analysis, the irreducible representation Г = 4A_1_ + 4E was obtained, which corresponds to the vibrational spectrum of the crystal in the center of the Brillouin zone. It follows from the theoretical group analysis that the vibrational spectrum of the AgAlS_2_ crystal contains 12 vibrational modes. There are four A_1_ symmetry and four E symmetry modes. Figure 10 shows the phonon dispersion ω(q) of the investigated crystal, calculated using the GGA-PBE function within the framework of the DFPT method. The phonon spectrum of the crystal consists of 12 phonon branches, which is consistent with the symmetry analysis. The crystal lattice is characterized by vibrations in the frequency range of 0–460 cm^−1^. The dispersion of phonons is constructed at the following points of the Brillouin zone, as well as the band structure Г → A → H → K → Г → M → L → H. It can be seen in the figure that when the acoustic branches approach the center of the Brillouin zone, the oscillation frequency goes to zero (ω → 0). Acoustic vibrations are represented by three vibration modes, which are represented by two lines on the phonon dispersion ω(q). The lower branch is formed by oscillations of symmetry E, which are doubly degenerate oscillations. At higher frequencies, the acoustic branch corresponds to oscillation of the A1 type. Thus, the representation for acoustic vibrations is Г_aco_ = A_1_ + E, while for optical circuits, the representation is Г_opt_ = 3A_1_ + 3E. There are no imaginary modes in the vibrational spectrum, which indicates the dynamic stability of the crystal in this phase. Optical branches have significant dispersion and interact with acoustic branches. Such a feature indicates a significant interaction between the constituent structural elements.

**Figure 11:**
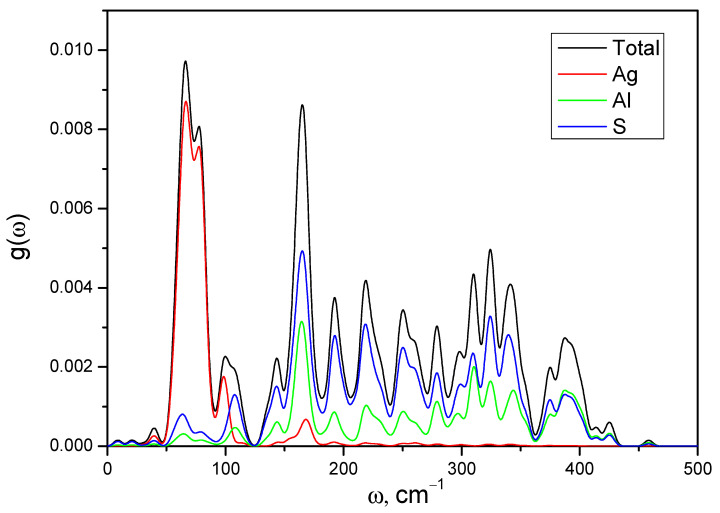
Calculated phonon density of states *N*(ω) of AgAlS_2_ crystals received using the linear response method together with the GGA-PBE function.

Figure 11 shows the calculated total and partial densities of the phonon states. The density of states consists of a multitude of narrow bands that overlap to form a wide band spanning frequency from 0 to 460 cm^−1^. From the consideration of the partial density of phonon states, it can be seen that low-frequency oscillations in the range of 50–100 cm^−1^ are formed by heavy silver atoms, Ag, interacting with sulfur atoms. Higher frequency oscillations are formed by Al and S atomic vibrations, which interact strongly over a wide frequency range, mainly contributing to the total density from 100 to 460 cm^−1^.

As one can see, the AgAlS_2_ crystal undergoes significant transformation compared to its state in the chalcopyrite crystal structure [12]. Crystal compression leads to a significant increase in phonon branch dispersion. 

**Figure 12:**
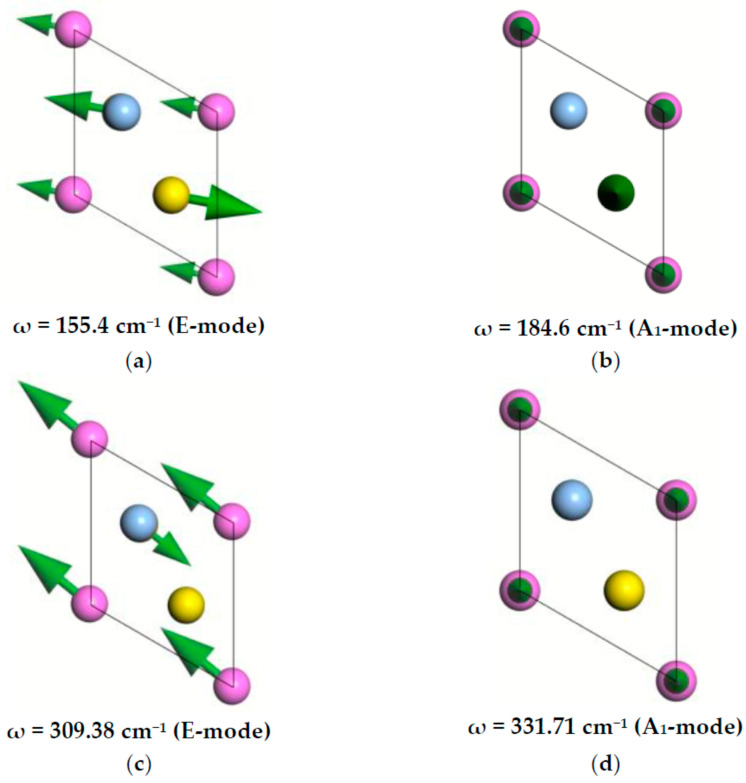
Vibrations in the AgAlS_2_ crystal in the high-pressure phase: (**a**,**c**) A_1_ symmetry; (**b**,**d**) E symmetry. Ag atoms are blue, Al atoms are purple, and S atoms are yellow spheres.

Table 6 lists the vibrational frequencies for the AgAlS_2_ crystal in phase II. It also presents the activity of vibrational modes in the IR and Raman spectra, as well as their irreducible representation. Figure 12 shows the types of atomic oscillations in the crystal lattice of the crystal under study for selected frequencies. Green arrows indicate the direction of vibration of the corresponding atoms. In the figure, blue balls correspond to Ag atoms, yellow corresponds to S atoms and purple corresponds to Al atoms.

**Figure 13:**
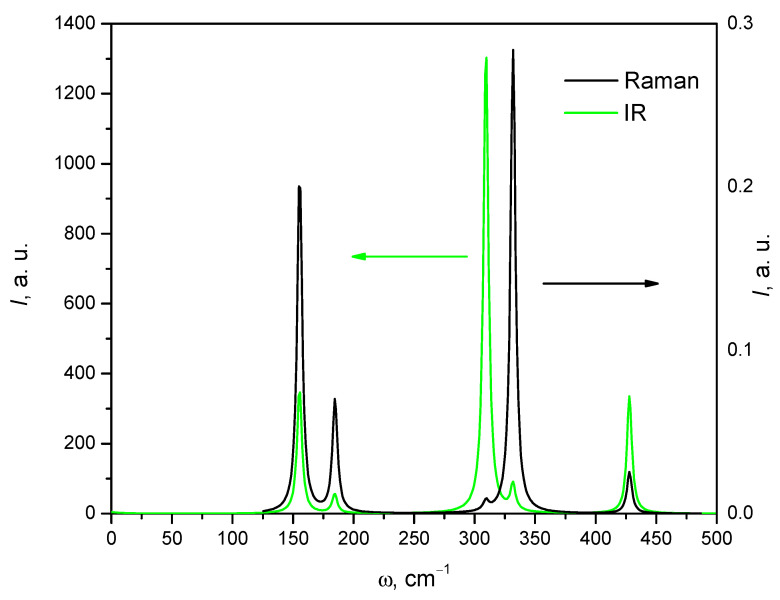
Calculated infrared spectra and Raman spectra for the AgAlS_2_ crystal in the high-pressure phase obtained using the GGA-PBE function.

Figure 13 shows the calculated infrared spectrum and the Raman spectrum of the AgAlS_2_ crystal in phase II. As can be seen in the figure, the spectrum of both IR and Raman is characterized by sets of characteristic bands of high intensity.

**Figure 14:**
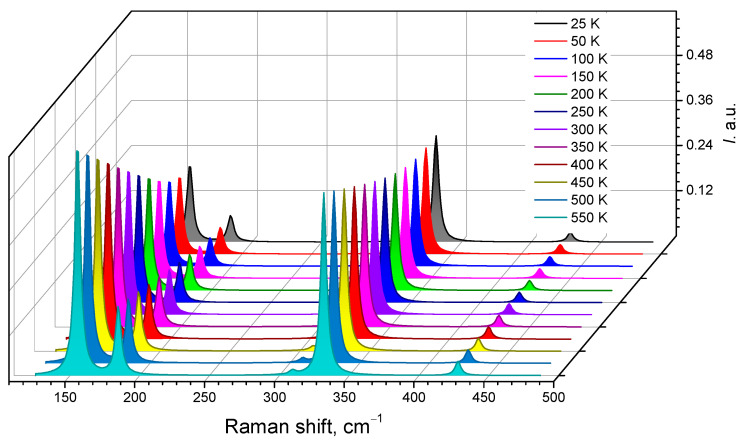
Calculated Raman spectra for the AgAlS_2_ crystal in the high-pressure phase obtained using the GGA-PBE function at different temperatures.

Figure 14 also presents the Raman spectra of the crystal for different temperatures in the range of 25–550K and λ_exc_ = 632.8 nm. As can be seen in the figure, the intensity of the band decreases as the temperature changes. For the band near 150 cm^−1^, the decrease in intensity is the largest compared to the other modes.

Unfortunately, we are not aware of any results regarding the study of the physical properties of the AgAlS_2_ crystal at high pressures. In particular, there are no studies of the vibrational states of this crystal. Therefore, the results obtained in the work may be useful for future experiments, predicting possible properties of the AgAlS_2_ crystal at high pressures.

## 4. Discussion

In this work, the structure and properties of the AgAlS_2_ crystal in structural phase II under high pressures were studied for the first time. A detailed crystal chemical analysis of the crystal structure of the AgAlS_2_ compound in known phases was carried out. We found that during the phase transition from a chalcopyrite-type structure to a trigonal structure, the second coordination sphere of sulfur atoms transitions from a cuboctahedron to a hexagonal analog, pointing toward a predominantly ionic type of chemical bonding within these ternary compounds. Our density functional theory computations revealed that the AgAlS_2_ crystal in the high-pressure phase exhibits the smallest indirect band gap of *E_g_* = 1.67 eV, with electronic transitions occurring from the top of the valence band to the bottom of the conduction band, corresponding to the M → Г transition. At the extremum points of the bottom levels of the conduction zone and the valence zone, the effective masses of the charge carriers are determined. Thus, for the *x* direction, m_e_* = 0.824 and m_h_* = 3.661, and for the *z* direction, m_e_* = 0.617 and m_h_* = 4.069 (in *m_e_* units). The effective mass of charge carriers has anisotropy in different directions, which is shown by the spatial distribution of the effective mass. The origin of the electronic levels was clarified using the calculations of the partial density of electronic states. The top of the valence band of the crystal is formed by 3*p*-states of sulfur with the contribution of 3*p*-states of aluminum. The bottom of the conduction zone is formed by *s*-states of Al atoms. Milliken charges and chemical bond populations were calculated, which showed an ionic covalent type of chemical bond with a large share of ionicity.

The real and imaginary parts of the dielectric function of the crystal were calculated. It is shown that the dielectric function takes slightly larger values for the AgAlS_2_ crystal in the chalcopyrite structure. The main characteristics of the dielectric function are similar to the chalcopyrite crystal, except for the absence of a well-defined band of low intensity at energies close to the band gap.

For the first time, we theoretically studied the vibrational properties of the AgAlS_2_ crystal. It was established that the vibration spectra consist of 12 vibrational modes. Three modes correspond to the acoustic branches, and nine modes are optical. It was revealed that the low-frequency vibrations are related to the Ag atoms, while the medium part of the DOS spectra is formed by the S atoms with the low contribution of the Ag and Al atoms. Theoretical infrared and Raman spectra for the AgAlS_2_ crystal are calculated for the first time.

## 5. Conclusions

In this work, we presented a comprehensive first-principle study of the high-pressure phase of the AgAlS_2_ crystal. The presented work is unique in its novelty and scope: the crystallographic, optical, vibrational, and electronic properties were studied. In all cases, results are in good agreement with experimental data.

We hope that the presented research will contribute to future development in the use of this material, which could include non-linear optics, water splitting, and photovoltaic applications. It can also serve as a reference point for the study of similar compounds that have comparable crystalline structure and chemical composition, as well as provide a useful methodology for further computational studies of related materials.

## Figures and Tables

**Table 1:** Structural parameters of the AgAlS_2_ crystal in the high-pressure phase represented by the *P*3*m*1 space group symmetry (space group No. 156) as determined by both experimental measurements [18] and calculations using the GGA-PBE function.

Parameter	Exp. [18]	GGA-PBE
*a*, Å	3.5	3.532
*b*, Å	3.5	3.532
*c*, Å	6.84	6.846
α, deg	90	90
β, deg	90	90
γ, deg	120	120
*V*, Å3	72.564	73.950
*Z*	1	1

**Table 2:** Optimized fractional atomic coordinates for the AgAlS_2_ crystal in the AgAlS_2_ crystal high-pressure phase as determined by both experimental measurements [18] and calculations using the GGA-PBE function. *D* is a difference between the calculated (optimized) and experimental fractional atomic coordinates *z*/*c* (all values are in Å).

	Exp. [18]			GGA-PBE			
	***x/a***	***y/b***	***z/c***	***x/a***	***y/b***	***z/c***	***D***
Ag	0.3333	0.6667	0.545	0.333333	0.666667	0.542616	0.0024
Al	0	0	0	0.000000	0.000000	0.008496	0.0085
S1	0.3333	0.6667	0.186	0.333333	0.666667	0.189055	0.0031
S2	0.6667	0.3333	0.814	0.666667	0.333333	0.804833	0.0092

**Table 3:** Atomic populations (by Mulliken) (in units of the proton charge) of the constituent atoms of the AgAlS_2_ crystal calculated using the GGA-PBE function.

Species	*s*	*p*	*d*	Total	Charge (e)	Hirshfeld Charge (e)
Ag	2.54	6.38	9.88	18.80	0.20	0.19
Al	0.88	1.43	0.00	2.31	0.69	0.20
S1	1.80	4.62	0.00	6.43	−0.43	−0.18
S2	1.82	4.64	0.00	6.46	−0.46	−0.21

**Table 4:** Lengths and overlap populations of the shortest atomic bonds and the bond ionicity of AgAlS_2_ crystal were calculated using the GGA-PBE function.

Bond	Population	Length (Å)	*f_h_*
Al—S1	1.34	2.38445	0.22
Al—S2	1.08	2.47016	0.07
Ag—S1	0.32	2.42060	0.88
Ag—S2	0.48	2.71667	0.66

**Table 5:** Character table of the AgAlS_2_ crystal in the high-pressure phase (II) in *C*_3*v*_ symmetry.

*C*_3*v*_	1	3	3	*m*	*m*	*m*
A_1_	1	1	1	1	1	1
A_2_	1	1	1	–1	–1	–1
E	2	–1	–1	0	0	0

**Table 6:** The frequencies of the vibrational modes are calculated for the AgAlS_2_ crystal in the high-pressure phase.

#	Irreducible Representation	Activity	Frequency, cm^−1^
1	A_1_	N/N	0
2	E	R/IR	0
3	E	R/IR	0
4	E	N/N	52.17
5	E	N/N	52.17
6	E	R/IR	155.45
7	E	R/IR	155.45
8	A_1_	R/IR	184.65
9	E	R/IR	309.38
10	E	R/IR	309.38
11	A_1_	R/IR	331.72
12	A_1_	R/IR	427.86

## Data Availability

All data will be made available on demand.
